# Integrative Transcriptome and Metabolome Analysis Identifies Potential Pathways Associated with Cadmium Tolerance in Two Maize Inbred Lines

**DOI:** 10.3390/plants14121853

**Published:** 2025-06-16

**Authors:** Pingxi Wang, Min Li, Xingye Ma, Bin Zhao, Xining Jin, Huaisheng Zhang, Shilin Chen, Xiangyuan Wu, Xiaoxiang Zhang

**Affiliations:** State Key Laboratory of Wheat-Maize Double Cropping and High-Efficiency Production, School of Agriculture, Henan Institute of Science and Technology, Xinxiang 453003, China; wangpingxi@hist.edu.cn (P.W.); 19915337953@163.com (M.L.); 18864781384@163.com (X.M.); zhaobin6662024@163.com (B.Z.); jinxining@126.com (X.J.); zhanghuaisheng1106@126.com (H.Z.); slchen@hist.edu.cn (S.C.)

**Keywords:** maize, cadmium stress, transcriptome, metabolome, seedling stage

## Abstract

Cadmium (Cd) significantly influences the morphological, physiological traits, and transport capacity of plants, but the underlying mechanism of Cd stress still remains to be further studied. In this study, physiological, transcriptomic, and metabolomic analyses were conducted to examine the morphological and physiological traits of two elite maize inbred lines, Chang7_2 (C7_2, a Cd-resistant line) and Zheng58 (Z58, a Cd-sensitive line) under control and Cd stress conditions. The results of morphological traits indicated that C7_2 reduced by 9.50–29.60% under Cd stress, whereas Z58 displayed more pronounced morphological changes ranging from 10.12 to 41.72% under Cd stress. Physiological assessments revealed that C7_2 maintained relatively stable antioxidant enzyme activity, while Z58 demonstrated more rapid alterations in the antioxidant system under Cd stress. Transcriptomic analysis identified 3030 differentially expressed genes (DEGs) unique to C7_2 and 4298 DEGs unique to Z58, with 1746 common DEGs shared between the two lines. Functional annotation revealed that the unique DEGs in C7_2 were mainly enriched in plant hormone signal transduction, plant–pathogen interactions, and the MAPK signaling pathway, while the unique DEGs in Z58 were mainly enriched in ribosome-related functions, plant hormone signal transduction, and phenylpropanoid biosynthesis. Metabolomic analysis identified 12 superclasses encompassing 896 metabolites in C7_2 and Z58, primarily including lipids and lipid-like molecules, organic acids and derivatives, as well as organoheterocyclic compounds. Analysis of differentially accumulated metabolites (DAMs) revealed fewer DAMs were accumulated in C7_2 under Cd stress. Further analysis identified that the three pathways of GPI anchor biosynthesis, glycerophospholipid metabolism, and purine metabolism were among the top 10 metabolic pathways in C7_2 and Z58. The integrative analysis highlighted the crucial roles of phenylpropanoid biosynthesis and zeatin biosynthesis in C7_2 for resistance to Cd stress. This study provides novel insights into the molecular and metabolic pathways underlying Cd tolerance in maize by integrating transcriptomic and metabolomic analyses of two contrasting inbred lines, providing a theoretical foundation for the future breeding of Cd-tolerant varieties.

## 1. Introduction

Cadmium (Cd) is a highly toxic heavy metal contaminant that is readily absorbed by plants due to its strong fluidity and solubility [1]. Cd not only disrupts the normal physiological metabolism of plants and diminishes crop quality, but it also poses a risk to human health through its transmission and accumulation in the food chain [2,3]. Cd stress inhibits plant growth, resulting in shortened leaf and root lengths as well as reduced leaf and root fresh weights [4,5]. Additionally, Cd stress can induce DNA damage, leading to alterations in the morphology and structure of cell membranes, mitochondria, and chloroplasts [6]. The activity of antioxidant enzymes, including superoxide dismutase (SOD), peroxidase (POD), and catalase (CAT), is also compromised under Cd stress, indicating an inhibition of antioxidant capacity [7]. Furthermore, the transport ability of Cd diminishes with higher concentrations and prolonged exposure to Cd stress, with Cd accumulation primarily occurring in the roots, followed by the stems, leaves, and fruits [8]. In a word, Cd significantly impacts the morphological, physiological characteristics, and transport capacity of plants, highlighting the necessity to investigate the underlying mechanisms of Cd stress.

In the past few years the research of Cd tolerance mechanism has been extended from physiological level to molecular regulation, and transcriptomic and metabolomic methods are employed to understand the molecular regulatory mechanism of Cd tolerance. Notably, a significant number of differentially expressed genes (DEGs) were identified under Cd stress, which involved in various physiological and biochemical processes including antioxidant defense, metal transport, and signal transduction [9,10,11]. Additionally, Cd was transported from sensitive tissues to non-sensitive tissues or excreted through metal transporters, and transcriptomic analysis elucidated the expression patterns of these metal transporter genes and their regulatory mechanisms [11,12]. Furthermore, Cd stress influences metabolic pathways at the metabolomic level, including amino acid metabolism, carbohydrate metabolism, and organic acid metabolism, leading to significant alterations in key metabolites within these pathways [13,14,15]. Cd stress can disrupt energy metabolism by impeding the synthesis of energy molecules such as adenosine triphosphate (ATP) [16]. Additionally, Cd stress may stimulate the production of secondary metabolites, which could mitigate toxic effects or play a role in signal transduction processes [4]. Through a comprehensive analysis of significantly altered genes and metabolites associated with the same biological processes, insights can be gained regarding the regulatory mechanism and the interaction between genes and metabolites [17,18]. This strategy has been applied in Kentucky bluegrass (*Poa pratensis*) [19], pepper (*Capsicum annuum* L.) [20], rice (*Oryza sativa* L.) [18], and tomato (*Solanum habrochaites*) [17]. These studies confirm that these two omics techniques are effective for understanding the mechanism of cadmium tolerance in other crops. In general, plants employ a multi-layered defense system against Cd toxicity, including transporter-mediated compartmentalization (e.g., ZIP, HMA, and NRAMP families for cellular influx/efflux; CAX and ABC transporters for vacuolar sequestration) and ligand-based chelation (e.g., glutathione, phytochelatins, organic acids, and metallothioneins) [8,12,16,21]. However, most insights derive from model species, and the integration of transcriptomic and metabolomic responses in maize—a globally vital crop with distinct genetic and physiological traits—remains underexplored. This study would bridge this gap by investigating Cd tolerance mechanisms in contrasting maize inbred lines, providing novel insights into crop-specific adaptive strategies. Roots serve as the primary site of Cd uptake and initial detoxification in plants, directly interfacing with soil contaminants [8,22]. While shoot responses are critical for systemic tolerance, root-specific mechanisms—such as metal sequestration, ligand synthesis, and transporter activity—are pivotal in mitigating Cd entry into the vascular system, thereby reducing shoot accumulation [16,21]. This study focuses on root-level adaptations to Cd stress, as these represent the first line of defense and a potential bottleneck for improving crop-wide Cd tolerance. In this study, two elite maize inbred lines (Chang7_2 (C7_2), a Cd-resistant line; Zheng58 (Z58), a Cd-sensitive line) were utilized as the research materials to investigate morphological and physiological traits. Additionally, transcriptomic and metabolomic analyses were conducted for the roots of C7_2 and Z58. The objectives of the study were as follows: (1) evaluate the effects of Cd stress on the morphological and physiological traits of C7_2 and Z58; (2) identify differentially expressed genes (DEGs), differentially accumulated metabolites (DAMs), and pathways associated with Cd stress in the two lines; and (3) establish a key regulatory network under Cd stress by integrating transcriptome and metabolome results. This research aims to elucidate the molecular and metabolic mechanisms underlying Cd tolerance in maize by integrating transcriptomic and metabolomic analyses of contrasting inbred lines, providing a theoretical foundation for future breeding of Cd-tolerant varieties.

## 2. Results

### 2.1. Effects of Cd Stress on Morphological and Physiological Changes for C7_2 and Z58

**Figure 1:**
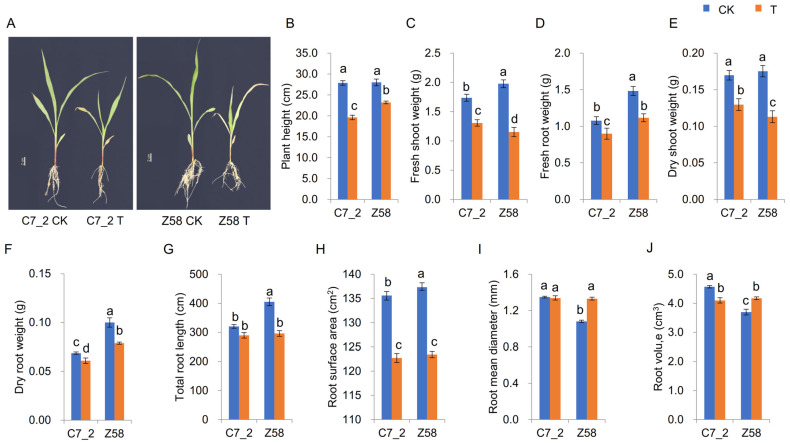
The morphological changes in C7_2 and Z58 under Cd stress. (**A**) Morphology of Z58 and C7_2 for 14-day-old seedlings. (**B**–**J**) Analysis of morphological traits for C7_2 and Z58. Different characters indicate significant differences at *p* value < 0.05 (n = 5).

The morphological traits of C7_2 and Z58 were examined under Cd stress (Figure 1A). In comparison to the CK group of C7_2, the plant height (PH), fresh shoot weight (FSW), fresh root weight (FRW), dry shoot weight (DSW), dry root weight (DRW), root surface area (RSA), and root volume (RV) of the Cd treatment were significantly reduced by 29.60%, 24.75%, 16.47%, 23.71%, 11.27%, 9.50%, and 10.28%, respectively (Figure 1B–F,H,J), while the total root length (TRL) and root mean diameter (RMD) did not show significant changes between the control and Cd stress treatment (Figure 1G,I). In the Z58 group, the PH, FSW, FRW, DSW, DRW, TRL, and RSA of the Cd treatment were significantly decreased compared to the control group by 17.14%, 41.72%, 24.76%, 35.55%, 21.20%, 26.93%, and 10.12%, respectively (Figure 1B–H), while the RMD and RV of the Cd treatment were increased significantly by 23.25% and 12.91% (Figure 1I,J). These results indicate that Z58 exhibited more pronounced morphological changes under Cd stress compared to C7_2, suggesting that Z58 is more sensitive to Cd stress than C7_2.

**Figure 2:**
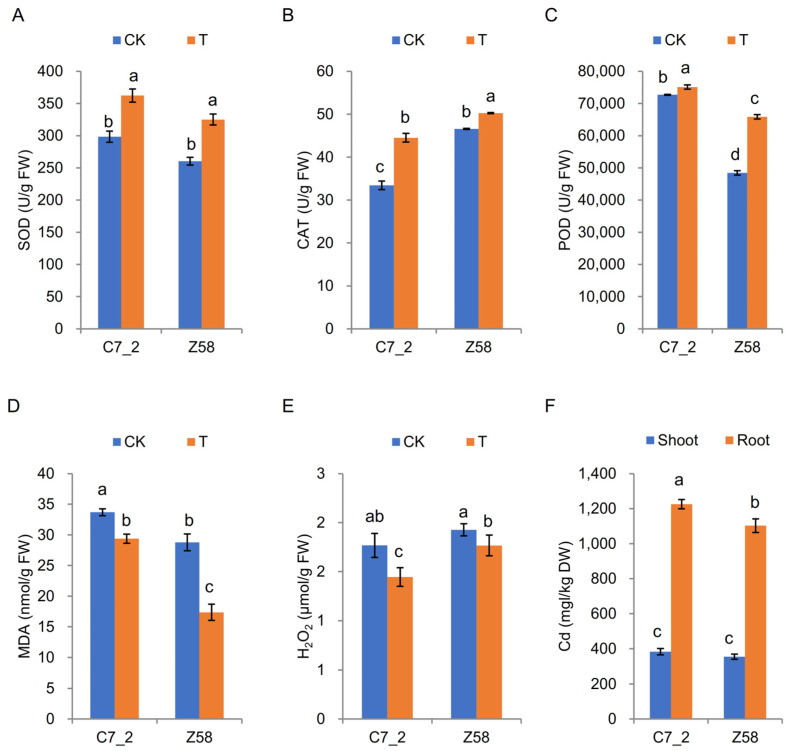
The SOD, POD, and CAT activity and MDA, H_2_O_2_, and Cd content changes in C7_2 and Z58 under Cd stress. (**A**) SOD. (**B**) POD. (**C**) CAT. (**D**) MDA. (**E**) H_2_O_2_. (**F**) Cd. The different characters indicate significant differences at *p* < 0.05 level (n = 5).

The physiological changes in C7_2 and Z58 were also examined under Cd stress (Figure 2A). In comparison to the control group of C7_2, the activities of SOD and CAT in the Cd stress treatment increased significantly by 21.36% and 33.11% (Figure 2A,B), the MDA and H_2_O_2_ content of the Cd treatment decreased significantly by 12.82% and 18.24% (Figure 2D,E), and POD activity remained unchanged under Cd stress (Figure 2C). For Z58, the activities of SOD, CAT and POD in the Cd stress treatment increased significantly by 24.79%, 7.90%, and 35.97%, respectively (Figure 2A–C), the MDA content in the Cd stress treatment was significantly decreased than that in CK by 39.69% (Figure 2D), whereas the H_2_O_2_ levels did not show significant changes under Cd stress (Figure 2E). Furthermore, the Cd content in the roots of C7_2 was significantly higher than that in Z58, but no significant differences were observed in the Cd content of the shoots between the two inbred lines (Figure 2F). The transfer coefficients from root to shoot for C7_2 and Z58 were 31.25% and 32.12%, respectively, indicating there was no significant difference for the transfer coefficients of C7_2 and Z58. These results suggest that Z58 exhibits relatively less stable antioxidant enzyme activity compared to C7_2, indicating that the antioxidant system in Z58 may be more severely disrupted under Cd stress.

### 2.2. The Transcriptomic Analysis of C7_2 and Z58 Under Cd Stress

#### 2.2.1. Analysis of RNA-Seq Data and DEGs for C7_2 and Z58 Under Cd Stress

**Figure 3:**
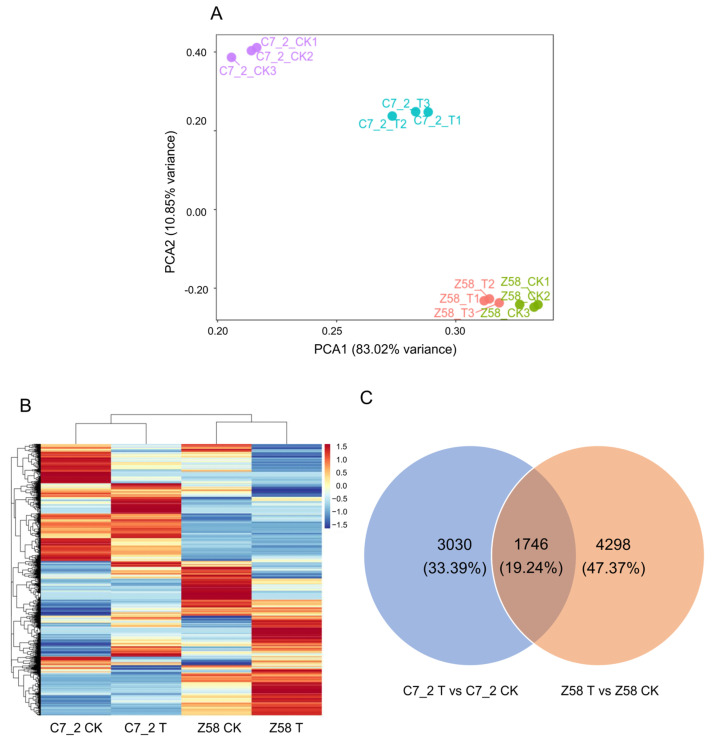
Analysis of transcriptome data for C7_2 and Z58 under CK and Cd stress. (**A**) PCA of the gene expression patterns of C7_2 and Z58 under CK and Cd treatment. The *X* axis represents PCA1 (83.02% variance), and the *Y* axis represents PCA2 (10.85% variance). Each sample has three biological duplicates and is represented on the plot by a unique symbol. (**B**) Heatmap of the gene expression patterns of C7_2 and Z58 under CK and Cd treatment. (**C**) Venn diagram of the DEGs in C7_2 T vs. C7_2 CK and Z58 T vs. Z58 CK.

To elucidate the response mechanisms under Cd stress, RNA-seq was conducted for 12 root samples from two lines of C7_2 and Z58, across two treatments and three replicates. As a result, a total of 71.62 Gb of clean data was obtained (Table S1). The Q20 values exceeded 99.95%, while the Q30 values ranged from 97.30% to 98.12%, confirming the reliability of the RNA-seq data (Table S1). Principal component analysis (PCA) was performed for the control (CK) and Cd treatment groups of C7_2 and Z58. PCA1 accounted for 83.02% of the total variability that separates samples by genotype (C7_2 vs. Z58), and PCA2 accounted for 10.85% that separates samples by treatment (Control vs. Cd) (Figure 3A). Heatmap analysis revealed distinct patterns of gene expression between C7_2 and Z58 under control and Cd stress treatment (Figure 3B).

The Venn diagram illustrates that 4776 DEGs (3774 up-regulated and 1002 down-regulated) were identified in C7_2, 6044 DEGs (2386 up-regulated and 3658 down-regulated) were identified in Z58, and 1746 (19.24%) common DEGs were shared between the two inbred lines (Figure 3C; Table S2). Transcriptomic analysis identified 2457 up-regulated and 573 down-regulated DEGs unique to the Cd-resistant line C7_2, while the Cd-sensitive line Z58 exhibited 1479 up-regulated and 2819 down-regulated unique DEGs under Cd stress (Figure 3C; Table S2). These results indicated that more up-regulated and less down-regulated genes were induced in C7_2 than that in Z58 under Cd stress.

#### 2.2.2. GO and KEGG Analysis for the DEGs of C7_2 and Z58 Under Cd Stress

**Figure 4:**
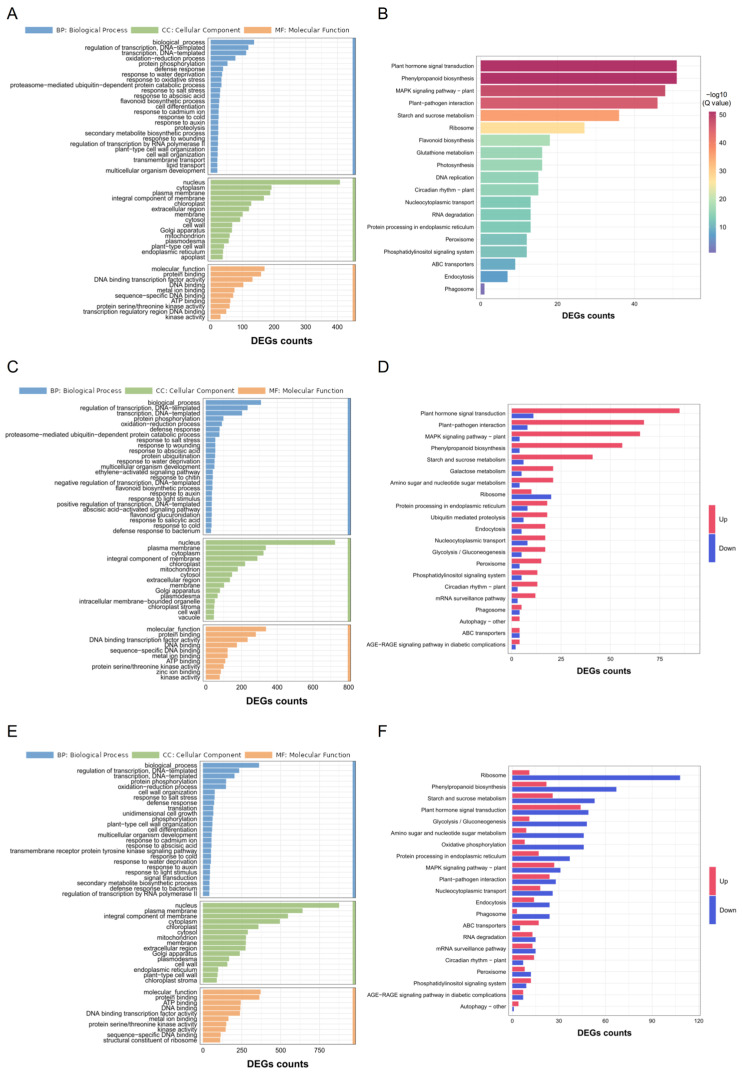
GO and KEGG_enrichment analysis for the DEGs of C7_2 and Z58 under Cd stress. (**A**) GO analysis for the 1746 common DEGs of C7_2 and Z58. (**B**) KEGG analysis for the 1746 common DEGs of C7_2 and Z58. (**C**) GO analysis for the 3030 unique DEGs in C7_2. (**D**) KEGG analysis for the 3030 unique DEGs in C7_2. (**E**) GO analysis for the 4298 unique DEGs in Z58. (**F**) KEGG analysis for the 4298 unique DEGs in Z58.

To further elucidate the roles of DEGs in response to Cd stress, GO and KEGG analysis were conducted for the common and unique DEGs of C7_2 and Z58. GO analysis for the 1746 common DEGs revealed significant enrichment in cellular component (CC) terms including the nucleus and cytoplasm, biological process (BP) terms such as regulation of transcription and oxidation–reduction processes, as well as molecular function (MF) terms including protein binding and DNA-binding transcription factor activity (Figure 4A). Similarly, KEGG analysis of these common DEGs indicated substantial enrichment in pathways related to plant hormone signal transduction, phenylpropanoid biosynthesis, the MAPK signaling pathway, plant–pathogen interactions, and starch and sucrose metabolism (Figure 4B; Table S3). In contrast, GO analysis of the unique DEGs in C7_2 and Z58 demonstrated enrichment in BP terms such as regulation of transcription, protein phosphorylation, and oxidation–reduction processes, as well as CC terms like the nucleus and plasma membrane, and MF terms including protein binding and DNA binding (Figure 4C,E). KEGG analysis for the unique DEGs in C7_2 revealed that those DEGs (2457 up-regulated and 573 down-regulated DEGs) were significantly enriched in 96 pathways, and plant hormone signal transduction, plant–pathogen interactions, the MAPK signaling pathway, phenylpropanoid biosynthesis, and starch and sucrose metabolism were ranked the top five pathways (Figure 4D; Tables S2 and S3). The unique DEGs (1479 up-regulated and 2819 down-regulated) in Z58 were significantly enriched in 107 pathways, and ribosome-related functions, plant hormone signal transduction, starch and sucrose metabolism, glycolysis/gluconeogenesis, and oxidative phosphorylation were ranked the top five pathways (Figure 4F; Tables S2 and S3). In summary, distinct molecular mechanisms responding to Cd stress existed between C7_2 and Z58.

#### 2.2.3. Construction of Traits-Associated Co-Expression Networks by WGCNA

**Figure 5:**
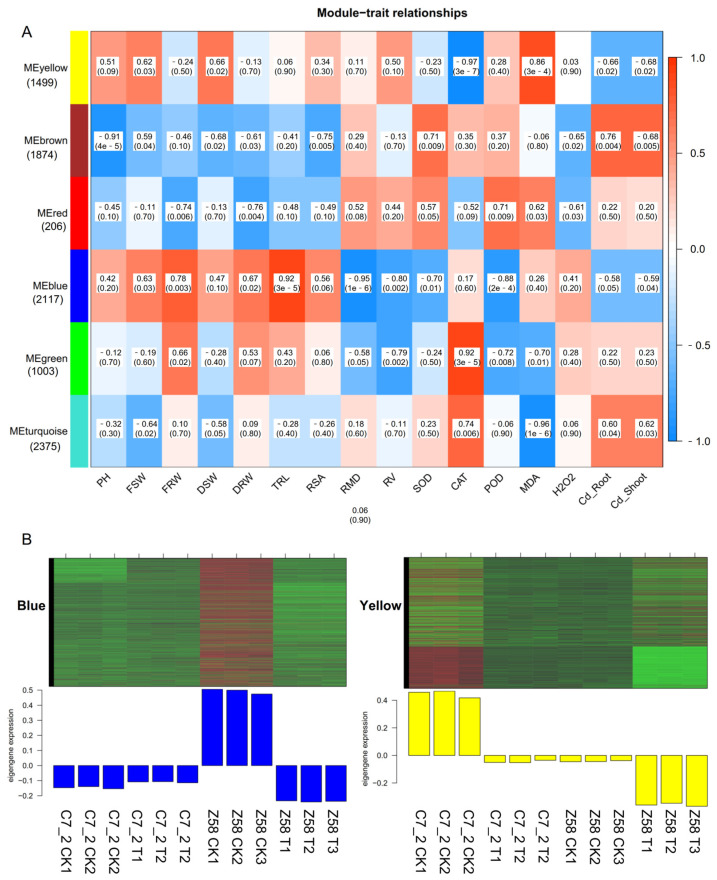
Identification of gene networks related to Cd stress in C7_2 and Z58. (**A**) Module–trait relationships of modules and the morphological and physiological traits. The horizontal coordinate represents different traits, the vertical coordinate represents different modules, and the filling value represents the correlation size and the statistical test *p* value. The closer the correlation value is to 1 or −1, the stronger the positive or negative correlation between the module and the samples. The smaller the *p* value in parentheses, the stronger the significance. (**B**) Heatmap of the blue and yellow module genes. The upper part is the heat map of gene expression within the modules, and the lower part is the bar plot of the expression of module eigengenes in each sample.

The DEGs in C7_2 and Z58 were analyzed using the WGCNA method, with morphological and physiological traits serving as clinical traits. As a result, six modules were identified (Figure 5A). The yellow module comprising 1499 DEGs was significantly associated with the physiological indicators of CAT (r = −0.97) and MDA (r = 0.86) (Figure 5A). The brown module, which included 1874 DEGs, showed a significant relationship with PH (r = −0.91). The blue module, containing 2117 DEGs, was significantly correlated with TRL (r = 0.92), RMD (r = −0.96) and POD (r = −0.88). The green module, consisting of 1003 DEGs, was significantly related to CAT. Furthermore, the turquoise module, which included 2375 DEGs, was significantly associated with MDA (r = −0.96). The blue and yellow modules were significantly related to more than two tested traits, and genes from these two modules were selected for further study. The DEGs of C7_2 in the blue and yellow modules were expressed relatively low compared to those in Z58 under Cd stress, respectively (Figure 5B). GO analysis revealed that the genes in the blue and yellow modules were primarily enriched in cellular components including the nucleus, plasma membrane, and cytoplasm (Figure S1A,C). KEGG analysis indicated that the genes in the blue module were mainly enriched in phenylpropanoid biosynthesis, starch and sucrose metabolism, and plant hormone signal transduction, whereas the genes in the yellow module were primarily enriched in ribosome function, plant hormone signal transduction, and plant–pathogen interactions (Figure S1B,D).

### 2.3. The Metabolomic Analysis Between C7_2 and Z58 Under Cd Stress

#### 2.3.1. PCA and Metabolite Analysis for C7_2 and Z58 Under Cd Stress

**Figure 6:**
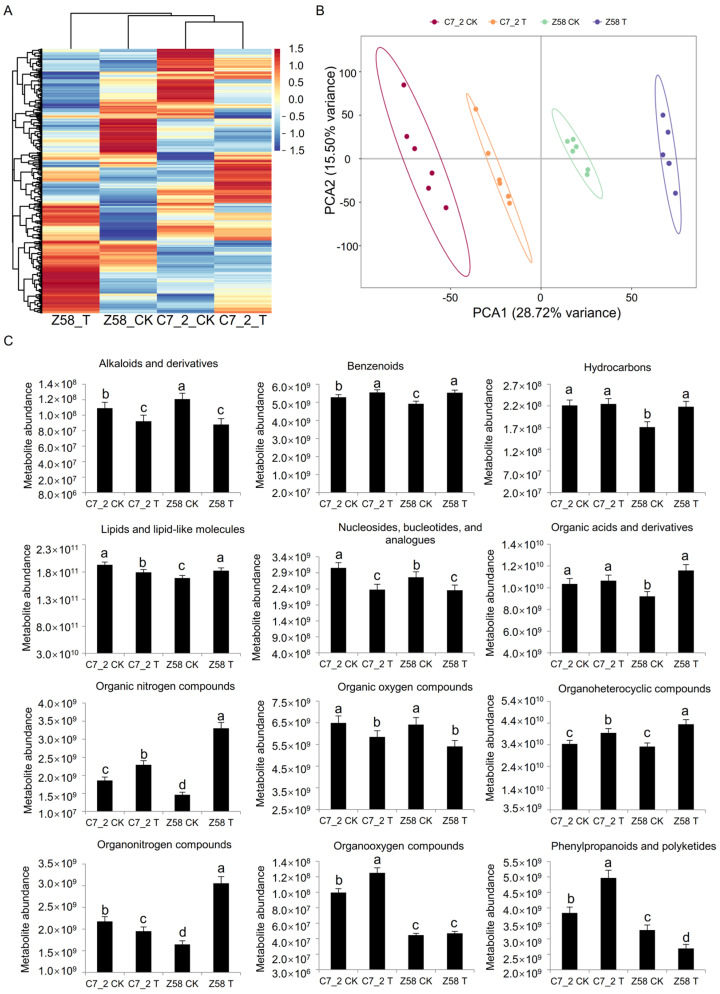
Analysis of metabolome data and metabolite content of C7_2 and Z58. (**A**) Cluster heat map analysis of DAMs. (**B**) Principal component analysis (PCA) of DAMs. (**C**) Differential metabolite content of 12 superclasses under Cd stress. The different characters indicate significant differences at a *p* < 0.05 level (n = 6).

To further elucidate the role of metabolites in the roots under Cd stress, a non-targeted metabolomics analysis was performed for 24 root samples from C7_2 and Z58 (2 lines × 2 treatments × 6 replicates). A hierarchical clustering heat map was constructed using standardized metabolite content (Figure 6A), revealing significant differences between C7_2 and Z58. PCA of the metabolites indicated that PCA1 accounted for 28.72% of the variability that separates samples by genotype (C7_2 vs. Z58), while PCA2 accounted for 15.50% (Figure 6B). A total of 896 metabolites were identified in C7_2 and Z58, which were categorized into 12 superclasses, including alkaloids and derivatives (0.45%), benzenoids (10.60%), hydrocarbons (1.45%), lipids and lipid-like molecules (36.38%), nucleosides, nucleotides, and analogues (2.79%), organic acids and derivatives (17.97%), organic nitrogen compounds (1.67%), organic oxygen compounds (6.70%), organoheterocyclic compounds (16.41%), organonitrogen compounds (0.67%), organooxygen compounds (0.67%), and phenylpropanoids and polyketides (4.24%) (Figure 6C). Under Cd stress, the metabolite abundance of benzenoids, hydrocarbons, organic acids and derivatives, organic nitrogen compounds, organoheterocyclic compounds, and organooxygen compounds increased in both C7_2 and Z58, whereas the abundance of alkaloids and derivatives, nucleosides, nucleotides, and analogues, as well as organic oxygen compounds, decreased. Notably, the abundance of hydrocarbons, lipids and lipid-like molecules, organonitrogen compounds, and phenylpropanoids and polyketides exhibited differing trends between C7_2 and Z58. Overall, the changes in metabolite abundance across the superclasses were gradual in C7_2, while Z58 displayed sharper fluctuations, particularly in the superclass of organic nitrogen compounds, suggesting that more metabolites were impacted in Z58.

#### 2.3.2. Venn and KEGG Analysis of the DAMs for C7_2 and Z58 Under Cd Stress

**Figure 7:**
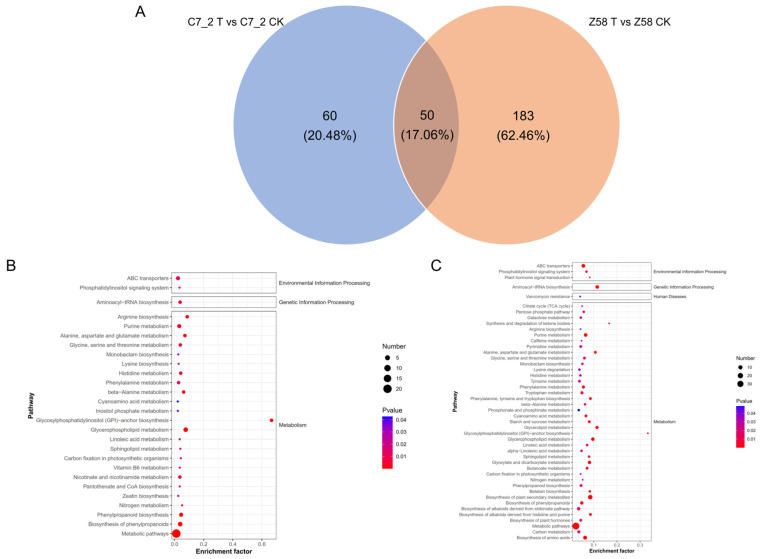
The Venn and KEGG pathway enrichment analysis for the DAMs of C7_2 and Z58. (**A**) Venn diagram analysis for the DAMs of C7_2 and Z58. (**B**) KEGG analysis for DAMs of C7_2 T vs. C7_2 CK. (**C**) KEGG analysis for DAMs of Z58 T vs. Z58 CK. The horizontal axis represents the enrichment ratio, while the vertical axis represents the enriched pathway name. The color scale indicates different thresholds of the *p* value, and the size of the dot indicates the number of metabolites corresponding to each pathway.

The Venn diagram of DAMs of C7_2 and Z58 reveals that 50 common DAMs were shared between the two lines, 60 DAMs (33 up-regulated and 27 down-regulated) were uniquely accumulated in C7_2, and 183 DAMs (132 up-regulated and 51 down-regulated) uniquely accumulated in Z58 (Figure 7A; Table S4). The results showed that fewer DAMs were accumulated in C7_2. Further analysis of the up-regulated DAMs in C7_2 and Z58 indicated that prenol lipids and benzene and its substituted derivatives constituted the majority of the DAMs.

KEGG pathway analysis revealed the DAMs of C7_2 were significantly enriched in 27 pathways, primarily in glycosylphosphatidylinositol (GPI) anchor biosynthesis, glycerophospholipid metabolism, metabolic pathways, arginine biosynthesis, and biosynthesis of phenylpropanoids (Figure 7B; Table S5). The DAMs of Z58 were significantly enriched in 46 pathways, primarily in the biosynthesis of plant secondary metabolites, aminoacyl-tRNA biosynthesis, metabolic pathways, the biosynthesis of amino acids, and glycerophospholipid metabolism (Figure 7C; Table S5). Furthermore, three pathways of GPI anchor biosynthesis, glycerophospholipid metabolism, and purine metabolism were all among the top 10 pathways in C7_2 and Z58, indicating these pathways play vital roles in responding to Cd stress.

### 2.4. Key Pathways Based on Joint Analysis of Transcriptome and Metabolism

**Figure 8:**
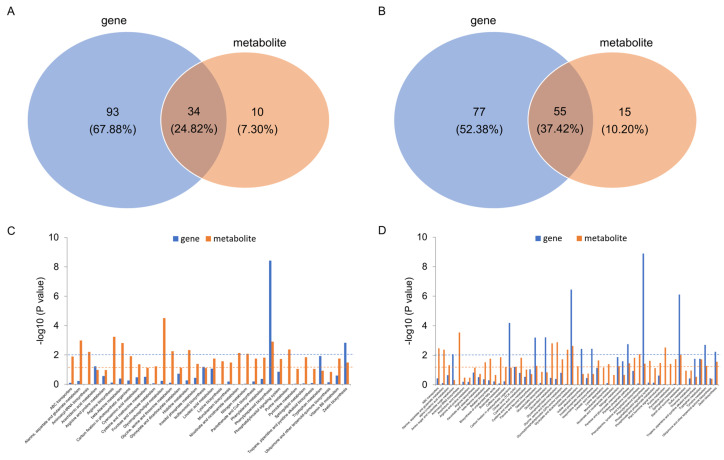
Analysis of KEGG enrichment pathways for the DEGs and DAMs of C7_2 and Z58. Venn diagram of KEGG enrichment pathways for the DEGs and DAMs of C7_2 (**A**) and Z58 (**B**). Bar plot of KEGG enrichment pathways for the DEGs and DAMs of C7_2 (**C**) and Z58 (**D**). The dashed blue and orange lines in (**C**,**D**) indicate the threshold value of KEGG enrichment pathways for DEGs and DAMs at *p* value < 0.01 and *p* value < 0.05, respectively.

The pathways of KEGG enrichment for the DEGs and DAMs of C7_2 and Z58 were conducted for joint analysis (Figure 8). As a result, 34 and 55 common KEGG pathways between DEGs and DAMs were identified in C7_2 and Z58, respectively (Figure 8A,B). Further examination of the 34 common KEGG pathways in C7_2 revealed that only two pathways exhibited significant changes in both DEGs and DAMs: phenylpropanoid biosynthesis and zeatin biosynthesis (Figure 8C). In contrast, among the 55 common KEGG pathways in Z58, 7 pathways displayed significant differences in both DEGs and DAMs, including carbon fixation in photosynthetic organisms, galactose metabolism, glyoxylate and dicarboxylate metabolism, the pentose phosphate pathway, phenylpropanoid biosynthesis, starch and sucrose metabolism, and tyrosine metabolism (Figure 8D). As a result, phenylpropanoid biosynthesis and zeatin biosynthesis were conducted for further study.

**Figure 9:**
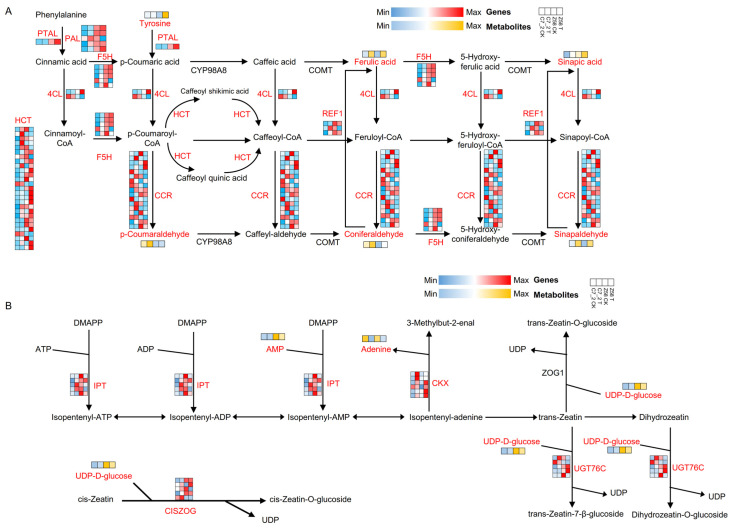
Integrated transcriptomic and metabolomic analysis for C7_2 and Z58 in phenylpropanoid biosynthesis and zeatin biosynthesis. (**A**) DEGs and DAMs of C7_2 and Z58 involved in phenylpropanoid biosynthesis. (**B**) DEGs and DAMs of C7_2 and Z58 involved in zeatin biosynthesis. The heatmap of (**A**,**B**) colored in blue and red indicates gene expression, and the heatmap of (**A**,**B**) colored in blue and orange indicates metabolite accumulation.

The gene expression levels of DEGs and metabolite content of DAMs were mapped to the pathways of phenylpropanoid biosynthesis and zeatin biosynthesis. In phenylpropanoid biosynthesis, 35 DEGs (31 up-regulated and 4 down-regulated) were identified in C7_2 under Cd stress, while 36 DEGs (15 up-regulated and 21 down-regulated) were identified in Z58 under Cd stress (Figure 9A). The genes predominantly included phenylalanine ammonia-lyase (PAL), phenylalanine/tyrosine ammonia-lyase (PTAL), 4-coumarate-CoA ligase (4CL), cinnamoyl-CoA reductase (CCR), ferulate-5-hydroxylase (F5H), shikimate O-hydroxycinnamoyltransferase (HCT), and coniferyl-aldehyde dehydrogenase (REF1) (Figure 9A). Additionally, six DAMs of C7_2 and Z58, including tyrosine, sinapic acid, ferulic acid, coniferaldehyde, p-coumaraldehyde, and sinapaldehyde, were identified in this pathway. Furthermore, the metabolite of coniferaldehyde was significantly up-regulated by 19.87-fold in Z58 under Cd stress, whereas no significant change was observed in C7_2. In contrast, the metabolite of sinapic acid was significantly up-regulated by 31.42-fold in C7_2 under Cd stress, while no significant change was noted in Z58 (Figure 9A). In zeatin biosynthesis, 13 DEGs (12 up-regulated and 1 down-regulated) were identified in C7_2 under Cd stress, while 13 DEGs (7 up-regulated and 6 down-regulated) were identified in Z58 under Cd stress (Figure 9B). The genes mainly included adenylate dimethylallyltransferase (IPT), cytokinin-N-glucosyltransferase (UTG76C), cytokinin dehydrogenase (CKX), and cis-zeatin O-glucosyltransferase (CISZOG) (Figure 9B). Furthermore, three DAMs of C7_2 and Z58 including adenine, UDP-D-glucose and AMP were identified in this pathway. The metabolites of UDP-D-glucose and AMP were down-regulated by 1.53-fold and 2.44-fold in Z58, but no significant change was observed in C7_2 (Figure 9B).

To verify the accuracy of RNA-seq data, 11 genes were selected for each key enzyme in the pathways of phenylpropanoid biosynthesis (seven key enzymes, such as PAL, PTAL, 4CL, CCR, F5H, HCT, and REF1) and zeatin biosynthesis (four key enzymes, such as IPT, UTG76C, CKS, and CISZOG) to perform qRT-PCR validation. The results revealed that the trends of qRT-PCR data were in accordance with the RNA-seq data, indicating the reliability of RNA-seq data (Figure S2).

## 3. Discussion

### 3.1. Effect of Cd Stress on the Morphological and Physiological Changes in C7_2 and Z58

Cd is a heavy metal characterized by high mobility and significant biological toxicity, and elevated levels of Cd contamination have severely disrupted ecosystems and pose substantial threats to plant growth and development [23,24]. Cd stress leads to reduced root size, decreased plant height, narrow and curly leaves, and abnormal morphological changes and alterations in root structure and surface area, ultimately diminishing the nutritional absorption capacity in plants [5]. Furthermore, Cd stress can result in stunted stems and adversely affect the development of leaf buds, inflorescences, and pollen, thereby directly impairing the reproductive capabilities of plants [11,25]. In this study, the measured traits including PH, FSW, FRW, DSW, DRW, and RSA were significantly decreased by 9.50–29.60% in C7_2 under Cd stress, while the traits were significantly decreased by 10.12–41.72% in Z58 under Cd stress (Figure 1B–F,H), indicating that normal growth was more severely impaired in Z58 under Cd stress. The TRL and RMD were significantly decreased by 26.93% and increased by 23.25% in Z58 under Cd stress, respectively, but these two traits showed no significant difference in C7_2 under Cd stress (Figure 1G,I). Furthermore, the RV exhibited contrasting trends in C7_2 and Z58 under Cd stress (Figure 1J). These results revealed that C7_2 exhibited greater tolerance to Cd stress.

Cd can form complex compounds with plant enzymes, proteins, nucleic acids, vitamins, and other essential substances, disrupting the normally metabolic processes of plants [8]. Cd stress can result in increased production of reactive oxygen species (ROS) in plants, causing direct damage to cell walls and membrane proteins, thus impairing the stability and permeability of plant cells [26]. Furthermore, Cd stress can alter the uneven distribution of Cd content within plants, disrupting cellular metabolism [27,28]. While Cd stress typically elevates ROS production, the resistant line C7_2 exhibited a significant decrease in H_2_O_2_ (18.24%, *p* < 0.05) and MDA (12.82%, *p* < 0.05) under Cd exposure (Figure 2D,E). This aligns with C7_2’s robust antioxidant response, where SOD and CAT activities increased significantly by 21.36% and 33.11%, respectively (Figure 2A,B), facilitating ROS scavenging and limiting lipid peroxidation. In contrast, the sensitive line Z58 showed a non-significant reduction in H_2_O_2_ (Figure 2E) and a marginal increase in CAT activity (7.9%, *p* < 0.05) under Cd stress, indicating inefficient H_2_O_2_ detoxification. Although MDA decreased sharply in Z58 (39.69%, *p* < 0.05), its significantly higher basal MDA (both under control and Cd stress) compared to C7_2 (Figure 2D) suggests chronic oxidative damage. Thus, Z58’s antioxidant system is less effective in maintaining redox homeostasis under Cd stress, resulting in pronounced physiological disruption. The stability of CAT activity in C7_2 mirrors findings in Cd-tolerant rice cultivars, where sustained catalase activity mitigated H_2_O_2_ accumulation [18]. In contrast, the erratic SOD/POD response in Z58 resembles stress-sensitive genotypes of wheat, where incomplete ROS scavenging exacerbated oxidative damage [28].

Despite the higher root Cd accumulation in C7_2 (Figure 2F), the similar transfer coefficients between C7_2 and Z58 (31.25% vs. 32.12%) suggest that root-to-shoot translocation is not the primary determinant of differential Cd tolerance. These results demonstrate that Cd^2+^ is predominantly distributed in the roots, aligning with findings from previous studies [22]. Instead, the stability of antioxidant systems (e.g., sustained CAT activity) and metabolic adjustments (e.g., phenylpropanoid up-regulation) in C7_2 may mitigate cellular damage, even under significant Cd flux. This underscores the need for multi-tissue omics and isotopic tracing to dissect compartment-specific detoxification strategies. Although our analysis was restricted to roots, the observed differences in Cd transfer coefficients (Figure 2F) and shoot Cd content between C7_2 and Z58 suggest that root-specific mechanisms (e.g., phenylpropanoid biosynthesis, antioxidant regulation) may indirectly influence shoot Cd dynamics. However, shoot-specific responses, such as vacuolar sequestration in leaf cells or phloem-mediated redistribution, likely contribute to the differential Cd tolerance observed and warrant future investigation.

### 3.2. Response of Transcriptome and Metabolome for C7_2 and Z58 Under Cd Stress

To further elucidate the potential response mechanisms of C7_2 and Z58 to Cd stress, RNA-seq was conducted on root samples from C7_2 and Z58 under control and Cd stress treatment. The predominance of up-regulated genes in C7_2 (e.g., phenylpropanoid biosynthesis, plant hormone signaling) suggests an active transcriptional reprogramming to counteract Cd stress, contrasting with the down-regulation of ribosome-related genes in Z58. This aligns with studies in rice and *Arabidopsis*, where Cd tolerance correlated with enhanced phenylpropanoid and hormone pathway activity [18,29]. These results indicated that more up-regulated and less down-regulated genes were induced in C7_2 than that in Z58 under Cd stress, indicating C7_2 engaged in a more robust response to cope with Cd stress, thus stimulated a series of defense mechanisms. KEGG analysis of the unique DEGs in C7_2 and Z58 revealed that plant hormone signal transduction, plant–pathogen interaction, MAPK signaling pathway—plant, phenylpropanoid biosynthesis, and starch and sucrose metabolism ranked the top five enrichment pathways in C7_2 (Figure 4D; Table S4), while these five pathways were ranked 2nd, 15th, 7th, 9th, and 3rd in Z58 according to their *p* values, respectively (Figure 4E,F; Table S4). Plant hormone signal transduction and starch and sucrose metabolism were both ranked the top five enrichment pathways in the unique DEGs of C7_2 and Z58. Plant hormone signal transduction primarily mitigates toxicity and enhances resistance to heavy metal stress by regulating gene expression, metabolic processes, and cell wall modifications [30,31,32]. Additionally, it reduces Cd accumulation and its associated toxicity in plants by modulating metabolic processes such as flavonoid biosynthesis, carbohydrate metabolism, and mitogen-activated protein kinase signaling pathways [32,33]. In starch and sucrose metabolism, an adequate energy supply and effective osmotic regulation are maintained under Cd stress, with regulation of resistance to Cd stress achieved through the expression of key enzymes such as sucrose synthetase and sucrose phosphorylase via signal transduction in plants [29,34]. In this study, the DEGs involved in the pathways of plant hormone signal transduction and starch and sucrose metabolism were mostly up-regulated in C7_2 under Cd stress, while they were mostly down-regulated in Z58 under Cd stress.

Roots as the most direct exposure to Cd stress, metabolite changes in the roots of C7_2 and Z58 were examined through non-target metabolome sequencing. A total of 896 metabolites across 12 superclasses were identified in C7_2 and Z58, with lipid and lipid-like molecules, organic acids and derivatives, and organoheterocyclic compounds comprising the majority of the identified metabolites (Figure 6C). Further analysis of the common DAMs up-regulated in C7_2 and Z58 indicated prenol lipids and benzene and its substituted derivatives constituted the majority of the DAMs (Table S5). Prenol lipids were of antioxidant activity and were able to remove reactive oxygen species and reduce oxidative damage in plants induced by heavy metal stress [35]. Benzene and its derivatives were also involved in the synthesis of lignin, which enhanced the mechanical strength and stability of cell walls and thus protected plants resisting abiotic stress [36]. KEGG pathway analysis for the DAMs of C7_2 and Z58 revealed three pathways of GPI anchor biosynthesis, glycerophospholipid metabolism, and purine metabolism were all among the top 10 pathways in C7_2 and Z58 (Figure 7B,C). In the pathway of GPI anchor biosynthesis, the barrier function of cell wall and membrane is enhanced by regulating the biosynthesis of GPI anchors [37]. The protein modified by GPI anchors may also participate in the signal transduction process in plants, causing changes in gene expression, the adjustment of metabolic pathways, and the remodeling of cell structure, thus adapting to resist the abiotic stress [38]. In glycerophospholipid metabolism, plants can maintain the fluidity and stability of cell membranes under stress by adjusting the synthesis and degradation rate of glycerophospholipids [39]. Furthermore, the metabolites of glycerophospholipid metabolism were related to antioxidant activity, which can remove reactive oxygen species and other oxidizing substances in plants and protect cells from oxidative stress [40]. In purine metabolism, a series of oxidative responses are induced to produce a substantial amount of ROS under Cd stress, which can interact with purine bases and their derivatives, leading to damage in DNA strands and proteins within plants [4]. To mitigate Cd stress in purine metabolism, the activity of antioxidant enzymes is enhanced, and the concentration of antioxidant substances increases, thereby facilitating the clearance of ROS and protecting cells from oxidative damage [41]. In this study, the metabolites associated with the GPI anchor biosynthesis, glycerophospholipid metabolism, and purine metabolism were up-regulated in C7_2 and Z58. Additionally, the activities of superoxide SOD, CAT, and POD were significantly increased, while the content of MDA was significantly decreased in both C7_2 and Z58.

A key limitation of this study is its exclusive focus on root tissues. While roots are central to Cd uptake and early detoxification, shoot-specific pathways—including photosynthetic adjustments, lignin deposition in stems, and phytohormone signaling in leaves—may further modulate Cd tolerance. Subsequent studies integrating shoot transcriptomics and metabolomics are essential to unravel systemic adaptation mechanisms. Validating root-derived candidates (e.g., PAL, 4CL) in shoot-specific contexts will clarify their roles in whole-plant Cd resistance.

### 3.3. Pathways of Phenylpropanoid Biosynthesis and Zeatin Biosynthesis Responding to Cd Stress in Maize

Phenylpropanoid biosynthesis is a critical metabolic pathway in plants, playing a vital role in growth, development, and defense mechanisms [42,43]. Lignin, a major component of the plant cell wall, contributes to structural stability and mechanical strength, thereby enhancing resistance to the penetration and toxicity of cadmium ions [44,45]. Additionally, phenolic compounds, such as sinapic acid, are known to exhibit antioxidant properties in plants under abiotic stress [46], though their direct role in Cd chelation in maize roots requires further investigation. These regulatory elements modulate the metabolites and products by influencing the expression and activity of key enzymes within the pathway [47,48]. As the first key enzyme in phenylpropane biosynthesis, any changes in PAL’s activity under Cd stress will directly impact the synthesis rate and yield of phenylpropane compounds [29]. The up-regulation of PAL and 4CL in C7_2 parallels observations in Cd-stressed Solanum habrochaites, where lignin biosynthesis reinforced cell walls to limit Cd influx [17]. However, unlike hyperaccumulators like Sedum alfredii, which sequester Cd via phenolic chelation [45], maize roots may prioritize lignin-mediated apoplastic barriers over direct Cd binding, as evidenced by high shoot translocation (Figure 2F). Notably, the metabolite of coniferaldehyde was significantly up-regulated by 19.87-fold in Z58 under Cd stress, while the metabolite of sinapic acid was significantly up-regulated by 31.42-fold in C7_2 under Cd stress. The metabolites of coniferaldehyde and sinapic acid in phenylpropanoid biosynthesis played key roles in the structural protection and functional maintenance of plants [43], the former primarily participated in the formation of lignin [49], while the latter was mainly involved in the synthesis of phenolic compounds and flavonoids [50]. These results indicate that distinct mechanisms exist in C7_2 and Z58 under Cd stress.

In zeatin biosynthesis, zeatin as a kind of plant cytokinin, it plays an important role in regulating the growth rate and physiological process in plants [51,52]. Zeatin can stimulate the division and proliferation of plant cells, thus promoting plant growth and development [53,54]. Furthermore, zeatin is also involved in regulating other physiological processes in plants, such as stomatal opening and closing, leaf expansion, and senescence [55,56]. The induction of IPT and CISZOG in C7_2 aligns with reports that cytokinins enhance Cd tolerance by stabilizing photosynthetic machinery and promoting root growth in Arabidopsis [52]. Conversely, the down-regulation of UDP-glucose and AMP in Z58 may reflect energy diversion toward stress mitigation, a phenomenon documented in Cd-sensitive barley genotypes [29]. UDP-D-glucose and AMP play an integral role in zeatin biosynthesis. UDP-D-glucose, a glycosyl donor and a basic substance for polysaccharide synthesis, contributes to the glycosylation of zeatin and polysaccharide synthesis in plants [57]. AMP, an energy transfer molecule and synthetic intermediate, is involved in biological processes such as energy metabolism, signal transduction, gene expression, and circadian clock regulation in plants [58]. Down-regulation of these two metabolites in zeatin biosynthesis will inevitably lead to the disturbance of energy metabolism and signal transmission in Z58, thus hindering the ability to resist heavy metal stress. These results suggest that phenylpropanoid biosynthesis and zeatin biosynthesis contribute to the enhanced resistance to Cd stress observed in C7_2.

The up-regulation of phenylpropanoid biosynthesis genes (e.g., PAL, 4CL) in C7_2 likely enhanced lignin synthesis (Figure 9A), reinforcing cell walls to limit Cd uptake. Concurrently, zeatin biosynthesis activation (e.g., IPT, CISZOG) promoted root growth and stress signaling, enabling resource reallocation to defense mechanisms. In contrast, Z58’s down-regulation of these pathways exacerbated Cd sensitivity, as evidenced by disrupted UDP-D-glucose and AMP levels (Figure 9B), which are critical for energy metabolism and stress adaptation. While our integrative analysis highlights phenylpropanoid and zeatin biosynthesis as pathways strongly associated with Cd tolerance in C7_2, these findings represent correlative relationships rather than causal evidence. For instance, although phenylpropanoid-related metabolites (e.g., sinapic acid) and genes (e.g., PAL, 4CL) were up-regulated in the Cd-tolerant line, direct experimental validation—such as enzymatic assays, gene knockout studies, or Cd-chelation experiments—is required to confirm their functional roles. Similarly, the observed reduction in Cd transfer to shoots in C7_2 (Figure 2F) suggests potential root sequestration mechanisms, but further work is needed to determine whether phenolic compounds directly participate in Cd binding or if other ligands (e.g., phytochelatins, organic acids) are primary contributors.

## 4. Materials and Methods

### 4.1. Plant Materials and Growth Conditions

According to a Cd stress resistance test conducted on an association panel of 350 diverse inbred lines in maize, two lines were selected for this study: the Cd-resistant line C7_2 and the Cd-sensitive line Z58, which serve as the parental lines of Zhengdan958 that are widely cultivated in China [59]. A total of 200 uniformly sized seeds from C7_2 and Z58 were disinfected using a 10% (*v*/*v*) NaClO solution (Sigma-Aldrich, St. Louis, MO, USA) for 20 min, followed by three washes with sterile water. The seeds were then germinated in plastic cups filled with vermiculite in a greenhouse for eight days, with temperature and light–dark cycles set at 28 °C/22 °C and 14 h/10 h, respectively. Subsequently, 120 seedlings with uniform size at the two-leaf stage were transplanted into 10 L plastic containers (30-hole cover plates and two seedlings per hole) filled with 1/2 modified Hoagland solution for three days. Subsequently, the 120 seedlings were treated with modified Hoagland solutions containing 0 mg/L (control, CK) and 20 mg/L (treatment, T; the concentration was confirmed by the pretreatment to maintain the normal growth of seedlings and also show distinct phenotypes under Cd stress between the two maize inbred lines) concentrations of CdCl_2_·2.5H_2_O solutions (Sigma-Aldrich, St. Louis, MO, USA) for seven days. The morphological and physiological traits of the seedlings for C7_2 and Z58 were then assessed, and the root samples of C7_2 and Z58 were collected for transcriptome and metabolome sequencing. Each treatment included three biological replicates, with ten plants per replicate.

### 4.2. Identification of Morphological and Physiological Traits

Five plants were randomly selected from each of the three biological replicates of C7_2 and Z58. The morphological traits investigated included PH, FSW, DSW, FRW, and DRW. The roots of C7_2 and Z58 were scanned using a root scanner (HED-GX, Horde·Electric, Hangzhou, China), and relevant root traits such as TRL, RSA, RMD, and RV were analyzed using the FG-RIAS root analysis software (v2.0, Horde Electric, Hangzhou, China). Root and shoot tissues were rinsed three times with deionized water to remove surface contaminants, oven-dried at 70 °C for 48 h, and ground into a fine powder. Approximately 0.2 g of dried tissue was digested in a mixture of 5 mL HNO_3_ (69%) and 2 mL H_2_O_2_ (30%) using a microwave digestion system (Mars 6, CEM Corporation, USA) at 180 °C for 15 min. Digested samples were diluted to 50 mL with deionized water, filtered through a 0.45 μm membrane, and analyzed for Cd content via an ICP-MS equipment (NexION^®^5000, PerkinElmer, Waltham, MA, USA). All kits (#BC4785 for CAT, #BC5165 for SOD, #BC0095 for POD, #BC0025 for MDA, #BC3595 for H_2_O_2_) were procured from Nanjing Ruoyuan Biotechnology Co., Ltd. (Nanjing, China), following the manufacturers’ protocols. In detail, SOD activity was determined by measuring the inhibition of nitroblue tetrazolium (NBT) reduction at 560 nm. One unit of SOD activity was defined as the amount of enzyme required to inhibit 50% of NBT reduction. POD activity was assayed via guaiacol oxidation at 470 nm, with one unit defined as the amount of enzyme catalyzing a 0.01 absorbance change per minute. CAT activity was quantified by monitoring H_2_O_2_ decomposition at 240 nm (ε = 39.4 mM^−1^cm^−1^), with one unit representing 1 μmol H_2_O_2_ decomposed per minute. MDA levels were measured using thiobarbituric acid (TBA) reaction at 532 nm, with results expressed as nmol/g FW based on an extinction coefficient of 155 mM^−1^cm^−1^. H_2_O_2_ was quantified via the titanium sulfate method at 415 nm, using a standard curve (0–100 μM H_2_O_2_).

### 4.3. RNA Extraction and Transcriptomic Analysis

Total RNA was extracted from 12 root samples of C7_2 and Z58 (2 lines × 2 treatments × 3 replicates) under control and Cd stress treatment using Trizol reagent (Thermofisher, Waltham, MA, USA, #15596018) according to the manufacturer’s instructions. Three replicates were conducted for each treatment. The RNA was reverse-transcribed into cDNA using the TransScript II One-Step gDNA Removal and cDNA Synthesis SuperMix kit (TransGen Biotech, Beijing, China) following the protocol of the manufacturer, and the quantitative real-time PCR (qRT-PCR) was conducted in a 7300 Sequence Detection System machine (Applied Biosystems, Foster City, CA, USA) using TransStart Green qPCR SuperMix (TransGen Biotech, Beijing, China). The primers used are presented in Table S6. *ZmGAPDH* was employed as the reference control.

Furthermore, the quantity and purity of the total RNA were assessed using a Bioanalyzer 2100 (Agilent, Santa Clara, CA, USA), and only high-quality RNA samples with an RIN number greater than 7.0 were utilized for sequencing library construction. Paired-end sequencing (PE150) was performed using an Illumina Novaseq™ 6000 (Illumina, San Diego, CA, USA) following the vendor’s recommended protocol. Differentially expressed genes (DEGs) between groups were analyzed using DESeq2 R package (v1.40.2). Genes were classified as DEGs if they met the criteria of a false discovery rate (FDR) of less than 0.05 and an absolute fold change (FC) of 2 or greater. The transcriptome analysis was based on B73 reference genome version 4 (https://www.maizegdb.org/). The DEGs of C7_2 and Z58 were conducted to functional enrichment analysis using the Gene Ontology (GO) database and the Kyoto Encyclopedia of Genes and Genomes (KEGG) pathway, facilitated by the OmicStudio tools (https://www.omicstudio.cn/tool, assessed on 18 June 2021).

Co-expression analysis was conducted using the weighted gene correlation network analysis (WGCNA) package (v1.72.1) in R (v4.3.1), following the guidelines outlined in published tutorials [60]. Hierarchical clustering of the root samples from C7_2 and Z58 was performed based on Euclidean distance computed from gene expression data, which was then integrated with the morphological and physiological traits of both C7_2 and Z58. Network topology analysis confirmed a scale-free topology network with a defined soft-thresholding power of 0.85. Modules were identified using the dynamic tree cutting algorithm, with parameters set for a minimum module size of 30 and a merge cut height of 0.25. For each module, the eigengene (the first principal component of the gene expressions within the module) was determined, and the correlations between eigengenes and the morphological and physiological traits were subsequently calculated.

### 4.4. Metabolite Extraction and Metabolomic Analysis

The 24 root samples of C7_2 and Z58 (2 lines × 2 treatments × 6 replicates) under control and Cd treatment were thawed on ice, and metabolites were extracted using 80% methanol (*v*/*v*, prepared by mixing 80 mL of HPLC-grade methanol with 20 mL of ultrapure water) containing 0.1% formic acid as a stabilizer. The extracted metabolites were subsequently sent for analysis at LC-Bio Technology Co., Ltd. (Hangzhou, China), with six replicates performed for each treatment. The acquired mass spectrometry (MS) data underwent several pretreatment steps, including peak picking, peak grouping, retention time correction, secondary peak grouping, and the annotation of isotopes and adducts, all conducted using XCMS software (v3.20.0). The LC-MS raw data files were converted into mzXML format and processed with the XCMS (v3.20.0), CAMERA(v1.56.0), and metaX (v1.28.0) toolboxes implemented in R software (v4.3.1). Each ion was identified by correlating the retention time (RT) with *m*/*z* data. The intensities of each peak were recorded, resulting in a three-dimensional matrix that contained arbitrarily assigned peak indices (retention time–*m*/*z* pairs), sample names (observations), and ion intensity information (variables). The online KEGG and HMDB databases were utilized to annotate the metabolites by matching the exact molecular mass data (*m*/*z*) of the samples with those in the databases. The intensity of peak data was further preprocessed using metaX. Features detected in fewer than 50% of quality control (QC) samples or 80% of biological samples were removed, and remaining peaks with missing values were imputed using the k-nearest neighbor algorithm to enhance data quality. Principal component analysis (PCA) was performed for outlier detection and evaluation of batch effects using the preprocessed dataset. Metabolites with a ratio ≥ 2.0 and VIP ≥ 1, or a ratio ≤ 0.5 and VIP ≥ 1, were classified as differentially accumulated metabolites (DAMs). Subsequently, the DAMs were performed for GO function and KEGG pathway analysis using the OmicStudio tools (https://www.omicstudio.cn/tool, assessed on 18 June 2021).

### 4.5. Statistical Analysis

The analyses were conducted using Microsoft Office 2010 (Microsoft, Redmond, WA, USA) and SPSS 20.0 (IBM, Armonk, NY, USA). Data are presented as mean ± standard deviation (SD), and significant differences were determined by two-way analysis of variance (ANOVA) at *p* value < 0.05. Multiple range tests were performed using Duncan’s method.

## 5. Conclusions

This study employs a joint transcriptome and metabolome analysis to elucidate the potential mechanisms by which C7_2 and Z58 respond to Cd stress. The results indicated that C7_2 exhibited greater tolerance to Cd compared to Z58, characterized by more stable antioxidant enzyme activity. Transcriptomic analysis revealed that key genes in C7_2 were regulated in response to Cd stress, primarily involving pathways associated with plant hormone signal transduction, plant–pathogen interactions, MAPK signaling, phenylpropanoid biosynthesis, and starch and sucrose metabolism. Furthermore, metabolome analysis identified the up-regulated metabolites in C7_2 under Cd stress, particularly prenol lipids, benzene and its substituted derivatives, as well as carboxylic acids and their derivatives. The integrative analysis highlighted the significant roles of phenylpropanoid biosynthesis and zeatin biosynthesis in conferring resistance to Cd stress in C7_2. This study identifies phenylpropanoid and zeatin biosynthesis as promising pathways underlying Cd tolerance in maize, offering a foundation for targeted functional studies. By integrating transcriptomic and metabolomic data, we provide a roadmap for validating candidate genes (e.g., PAL, 4CL, IPT) and engineering Cd-resilient maize varieties through breeding or metabolic modulation.

## Data Availability

All data that support the findings of this study are included in this manuscript and its Supplementary Information files. Further inquiries can be directed to the corresponding author(s).

## Supplementary Materials

The following supporting information can be downloaded at: https://www.mdpi.com/article/10.3390/plants14121853/s1, Figure S1. GO and KEGG enrichment analysis of the blue and yellow module. GO enrichment analysis for the blue (A) and yellow module (C). KEGG enrichment analysis for the blue (C) and yellow module (D). The *x*-axis represents Rich Factor of enrichment analysis, and the *y*-axis is the entry of the KEGG pathway. In the bubble diagram of (C,D), the size of the bubble represents the number of genes enriched, and the color of the bubble represents the *p* value of the enrichment analysis. Figure S2. Validation of the DEGs selected for the key enzymes in phenylpropanoid biosynthesis and through qRT-PCR. The *x*-axis represents the value of log2(fold change) for RNA-seq data, and the *y*-axis represents the value of log2(fold change) for qRT-PCR data. Table S1. Statistics of RNA-seq data of C7_2 and Z58. Table S2. The differentially expressed genes (DEGs) identified in C7_2 and Z58 under control and Cd stress. Table S3. KEGG pathway enrichment analysis for the DEGs identified in C7_2 and Z58 under control and Cd stress. Table S4. The differentially accumulated metabolites (DAMs) in C7_2 and Z58 under control and Cd stress. Table S5. KEGG pathway enrichment analysis for the DAMs in C7_2 and Z58 under control and Cd stress. Table S6. Primers used for qRT-PCR verification.
