# Supplementary figures and images for: Integrative Transcriptome and Metabolome Analysis Identifies Potential Pathways Associated with Cadmium Tolerance in Two Maize Inbred Lines
Source: Plants (Basel). 2025 Jun 16;14(12):1853. doi: 10.3390/plants14121853 (PMC12196682; doi:10.3390/plants14121853)

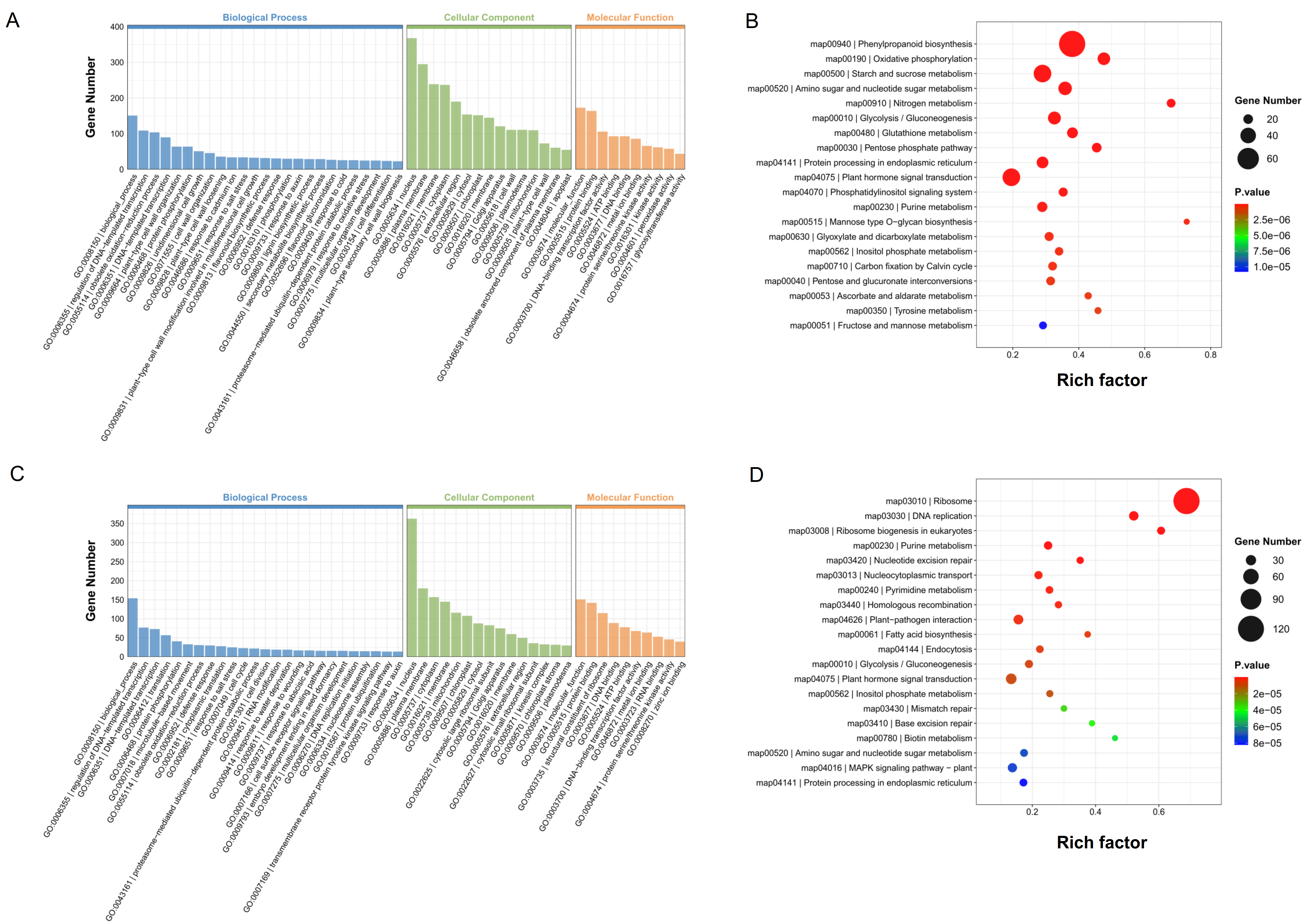

Supplement: Supplementary file 1 [file plants-14-01853-s001.zip › Figure S1.tif]

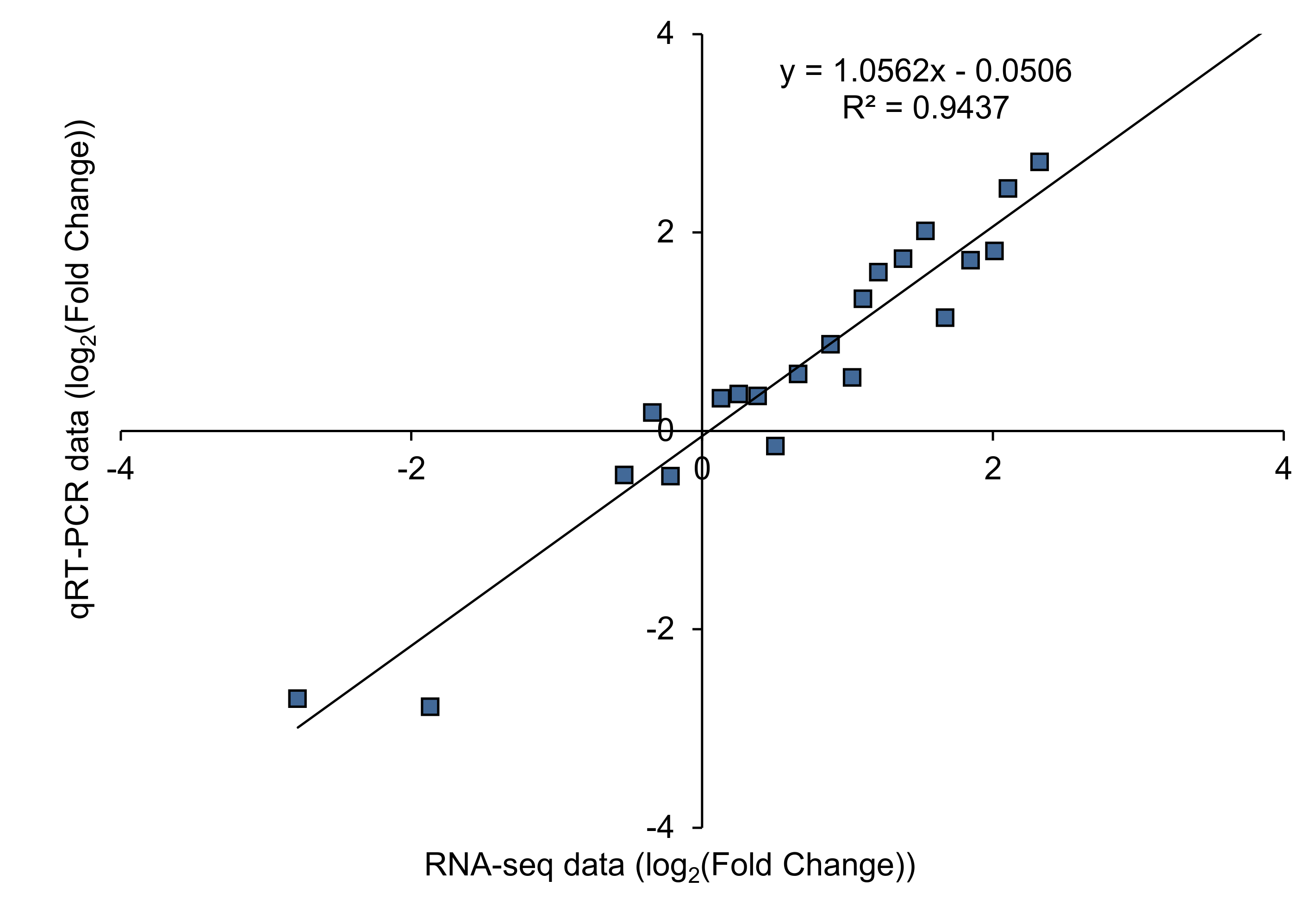

Supplement: Supplementary file 1 [file plants-14-01853-s001.zip › Figure S2.tif]
